# Irisin-loaded electrospun core-shell nanofibers as calvarial periosteum accelerate vascularized bone regeneration by activating the mitochondrial SIRT3 pathway

**DOI:** 10.1093/rb/rbad096

**Published:** 2023-10-31

**Authors:** Xi Hua, Mingzhuang Hou, Lei Deng, Nanning Lv, Yong Xu, Xuesong Zhu, Huilin Yang, Qin Shi, Hao Liu, Fan He

**Affiliations:** Department of Orthopaedics, The First Affiliated Hospital of Soochow University, Soochow University, Suzhou 215006, China; Orthopaedic Institute, Suzhou Medical College, Soochow University, Suzhou 215000, China; Department of Orthopedics, Suzhou Wuzhong People’s Hospital, Suzhou, Jiangsu Province 215128, China; Department of Orthopaedics, The First Affiliated Hospital of Soochow University, Soochow University, Suzhou 215006, China; Orthopaedic Institute, Suzhou Medical College, Soochow University, Suzhou 215000, China; Department of Orthopaedics, The First Affiliated Hospital of Soochow University, Soochow University, Suzhou 215006, China; Orthopaedic Institute, Suzhou Medical College, Soochow University, Suzhou 215000, China; Department of Orthopaedics, The First Affiliated Hospital of Soochow University, Soochow University, Suzhou 215006, China; Orthopaedic Institute, Suzhou Medical College, Soochow University, Suzhou 215000, China; Department of Orthopedic Surgery, Lianyungang Clinical College of Xuzhou Medical University, Lianyungang 222003, China; Department of Orthopaedics, The First Affiliated Hospital of Soochow University, Soochow University, Suzhou 215006, China; Orthopaedic Institute, Suzhou Medical College, Soochow University, Suzhou 215000, China; Department of Orthopaedics, The First Affiliated Hospital of Soochow University, Soochow University, Suzhou 215006, China; Orthopaedic Institute, Suzhou Medical College, Soochow University, Suzhou 215000, China; Department of Orthopaedics, The First Affiliated Hospital of Soochow University, Soochow University, Suzhou 215006, China; Orthopaedic Institute, Suzhou Medical College, Soochow University, Suzhou 215000, China; Department of Orthopaedics, The First Affiliated Hospital of Soochow University, Soochow University, Suzhou 215006, China; Orthopaedic Institute, Suzhou Medical College, Soochow University, Suzhou 215000, China; Department of Orthopaedics, The First Affiliated Hospital of Soochow University, Soochow University, Suzhou 215006, China; Orthopaedic Institute, Suzhou Medical College, Soochow University, Suzhou 215000, China; Department of Orthopaedics, The First Affiliated Hospital of Soochow University, Soochow University, Suzhou 215006, China; Orthopaedic Institute, Suzhou Medical College, Soochow University, Suzhou 215000, China

**Keywords:** periosteum, irisin, mitochondrial function, critical-sized bone defect, redox homeostasis

## Abstract

The scarcity of native periosteum poses a significant clinical barrier in the repair of critical-sized bone defects. The challenge of enhancing regenerative potential in bone healing is further compounded by oxidative stress at the fracture site. However, the introduction of artificial periosteum has demonstrated its ability to promote bone regeneration through the provision of appropriate mechanical support and controlled release of pro-osteogenic factors. In this study, a poly (l-lactic acid) (PLLA)/hyaluronic acid (HA)-based nanofibrous membrane was fabricated using the coaxial electrospinning technique. The incorporation of irisin into the core-shell structure of PLLA/HA nanofibers (PLLA/HA@Irisin) achieved its sustained release. *In vitro* experiments demonstrated that the PLLA/HA@Irisin membranes exhibited favorable biocompatibility. The osteogenic differentiation of bone marrow mesenchymal stem cells (BMMSCs) was improved by PLLA/HA@Irisin, as evidenced by a significant increase in alkaline phosphatase activity and matrix mineralization. Mechanistically, PLLA/HA@Irisin significantly enhanced the mitochondrial function of BMMSCs via the activation of the sirtuin 3 antioxidant pathway. To assess the therapeutic effectiveness, PLLA/HA@Irisin membranes were implanted *in situ* into critical-sized calvarial defects in rats. The results at 4 and 8 weeks post-surgery indicated that the implantation of PLLA/HA@Irisin exhibited superior efficacy in promoting vascularized bone formation, as demonstrated by the enhancement of bone matrix synthesis and the development of new blood vessels. The results of our study indicate that the electrospun PLLA/HA@Irisin nanofibers possess characteristics of a biomimetic periosteum, showing potential for effectively treating critical-sized bone defects by improving the mitochondrial function and maintaining redox homeostasis of BMMSCs.

## Graphical Abstract

**Figure:**
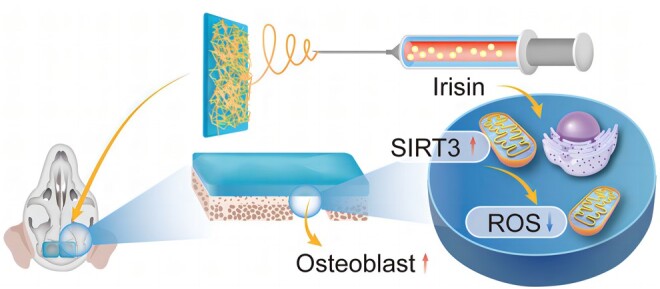

## Introduction

The repair of large segmental bone defects poses a formidable challenge for orthopedic surgeons due to the intricacy of the treatment, complex surgical procedures and a multitude of potential complications [1]. Over the past decades, several surgical interventions have been developed to treat bone defects, such as vascularized fibula transplantation [2], induced membrane technique (IMT) [3] and distraction osteogenesis [4]. Among these, the IMT has gained widespread application due to its autonomy from autologous bone transplantation and notable therapeutic efficacy, making it a primary treatment modality for large segment bone defects [5]. A recent clinical investigation demonstrated a success rate of 75% for multi-staged IMT in the treatment of patients with critical-sized bone defects (CSBDs) [6]. Another investigation reported that individuals afflicted with extensive bone defects were able to regain limb functionality and bear full weight after an average of 8.5 months following the implementation of IMT treatment [7]. The fundamental tenet of IMT involves the creation of a fresh periosteum on the bone surface, thereby expediting bone regeneration. Presently, the utilization of membranes for addressing bone defects is incapable of hastening bone healing due to their osteoinductive inability. Consequently, it is necessary to develop new biomimetic artificial periosteum for clinical applications.

The periosteum, a fibrous tissue that envelops the exterior of bone tissues, serves as a natural reservoir of multipotent progenitor cells that facilitate the mending of bone defects [8]. In the process of bone formation, the periosteum releases a diverse array of cytokines and triggers the osteogenic differentiation of stem cells, thereby expediting endochondral and intramembranous ossification [9]. Additionally, it has been demonstrated that the absence of periosteum inevitably leads to nonunion or malunion, whereas the transplantation of periosteum expedites the process of bone regeneration, given the crucial function of periosteum [10]. Consequently, there has been extensive research on bone tissue engineering, with a focus on developing biomimetic periosteum [11]. In recent years, electrospinning has emerged as a prominent method for fabricating two-dimensional (2D) nanofibrous membranes and gained great attraction in bone tissue engineering [12]. Electrospinning is a form of electrohydrodynamics technology, whereby a polymer solution is atomized into a fine jet through the application of high-voltage electricity [13]. The electrospun nanofibers exhibit morphological similarities to the natural extracellular matrix (ECM) and possess drug carrying and releasing capabilities [14]. Nevertheless, polymer-based electrospun membranes suffer from a deficiency in biological activity, so the incorporation of osteogenic or angiogenic factors is necessary.

Irisin, a cytokine secreted by muscle during exercise, was initially identified as a participant in the browning of white adipose tissue [15]. Its involvement in various diseases, such as insulin resistance, type II diabetes mellitus, and metabolic syndrome, has been established [16]. Recently, the impact of irisin in musculoskeletal disorders, in addition to its endocrine functions, has attracted substantial research interest. The administration of irisin has been demonstrated to have multifaceted benefits, including the amelioration of metabolic dysfunction and age-related sarcopenia, as well as the rescue of skeletal muscle mass loss induced by denervation [17]. These effects are achieved through the activation of myogenic differentiation in muscle-derived satellite stem cells and the prevention of myotube-related protein degradation [18]. Additionally, irisin has been shown to regulate bone formation and resorption by protecting osteocytes from apoptosis and inducing the expression of sclerostin *in vivo* [19]. Furthermore, irisin has been identified as an effective enhancer of mitochondrial function, according to several studies. The regulation of mitochondrial uncoupling protein 2 (UCP2) by irisin has been demonstrated to prevent ischemia/reperfusion (IR)-induced oxidative stress and preserve mitochondrial function [20]. Irisin has also been shown to increase mitochondrial content and promote the expression of mitochondrial transcription factor and peroxisome proliferative activated receptor-γ co-activator 1α (PGC-1α), both of which are related to mitochondrial biogenesis [21]. Based on this previous literature, we hypothesized that irisin-loaded electrospun nanofibers might be an excellent biomimetic periosteum to accelerate the healing of bone defects by enhancing the mitochondrial function of bone marrow-derived mesenchymal stem cells (BMMSCs).

**Figure 1.**
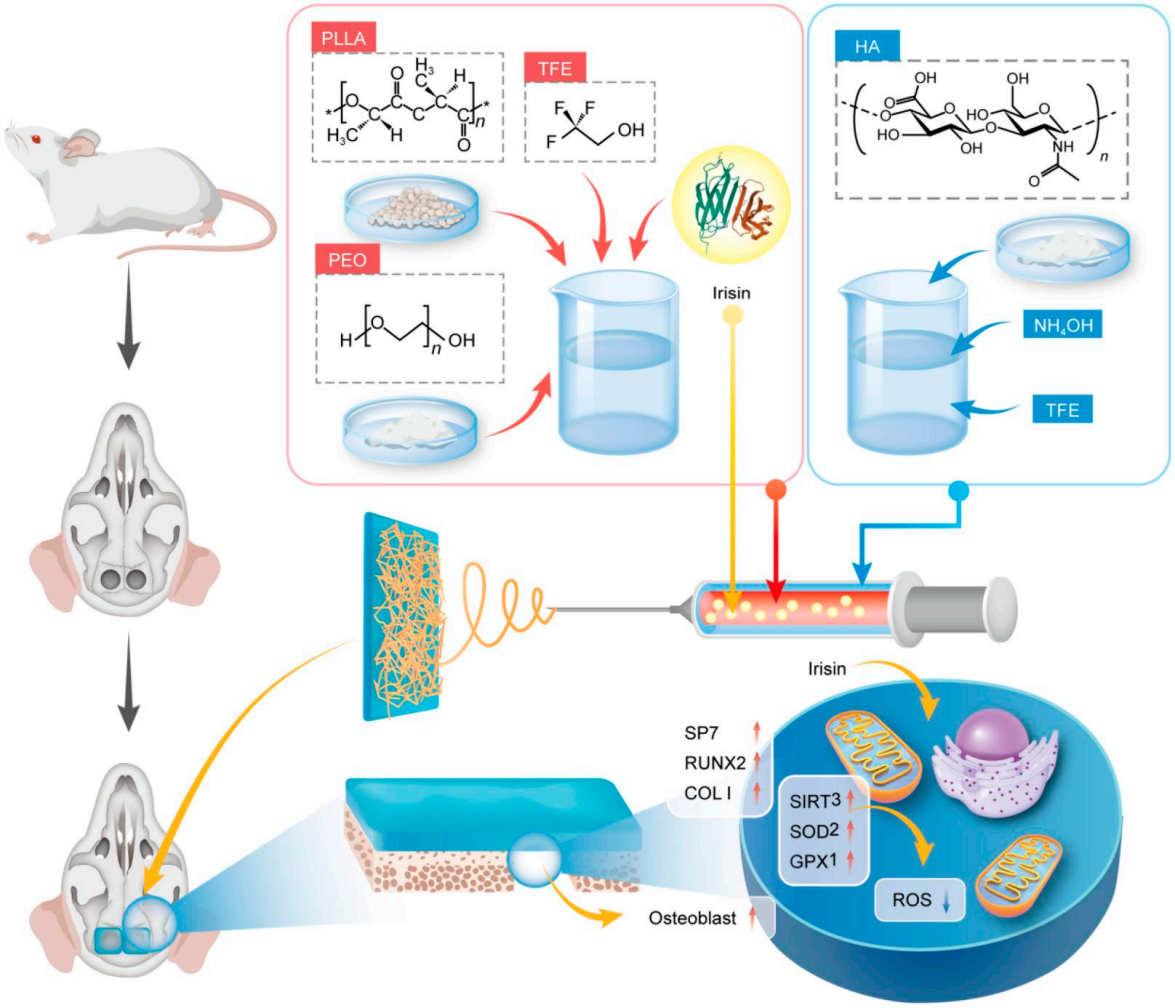
Schematic diagram of the preparation of biomimetic irisin-loaded electrospun nanofibers (PLLA/HA@Irisin) using coaxial electrospinning and the therapeutic application in a critical-sized calvarial defect.

The bionic nanofibrous periosteum was fabricated by employing the coaxial electrospinning technique, with a core layer of poly (l-lactic acid) (PLLA) loaded with irisin, and a shell layer consisting of sodium hyaluronate (HA). The performance of PLLA/HA@Irisin nanofibers was evaluated in terms of their mechanical properties and irisin-release ability, as well as the impact on the osteogenic differentiation, antioxidant capacity and mitochondrial function of BMMSCs. The therapeutic efficacy of PLLA/HA@Irisin nanofibers was subsequently investigated in a rat critical-sized calvarial defect model (Figure 1).

## Materials and methods

### Fabrication and characterization of PLLA/HA@Irisin membranes

#### Fabrication of PLLA/HA@Irisin membranes

PLLA was procured from Daigang Biomaterials (Jinan, China). Polyethylene oxide (PEO) was purchased from Avocado (London, UK). HA was obtained from Bloomage Freda Biopharma (Shandong, China). Dimethyl sulfoxide and trifluoroethanol (TFE) with a purity of ≥99.0% were acquired from Sigma-Aldrich (St Louis, MO, USA). The present study employed a stable jet coaxial electrospinning method to fabricate aligned core-shell nanofibers, utilizing two precision syringes as a coaxial jet device. The core and shell solutions were prepared by incorporating PEO (1% w/v)-doped PLLA (5% w/v) and HA (1% w/v)-doped TFE (NH_4_OH: TFE = 2:1, v/v) solvent mixtures, respectively. During the electrospinning process, a potential of 18 kV, consisting of a high-voltage power supply (Tianjin Dongwen High Voltage Power Supply Co., LTD., China), was applied to the coaxial electrospinning. Flow rate during electrospinning was set at 0.5 ml/h for the core and 0.2 ml/h for the shell. A ground roller rotating at 1000 rpm was used as the collector. After dissolving 0.5 g of PLLA and 0.1 g PEO in 5 ml of TFE, 0.1 and 1 μg of irisin were separately added to the PLLA solution, separately. Subsequently, electrospinning was conducted according to the steps described above. The spinning membranes were designated as PLLA/HA@Irisin(L) membrane containing a low concentration irisin at 20 mg/ml, and PLLA/HA@Irisin(H) membrane containing a high concentration irisin at 200 mg/ml.

#### Scanning electron microscope

In order to examine the microstructure morphology, the specimens were affixed to scanning electron microscope (SEM) sample stubs using conductive tape and subsequently coated with gold/palladium via an ion sputtering apparatus (SC7620, Quorum Technologies, East Sussex, UK). The morphology was subsequently analyzed using a SEM (S-4800, Hitachi, Tokyo, Japan) at an accelerated voltage of 15 kV.

#### Transmission electron microscope

Following the electrospinning process, the internal structure of the individual fibers was examined via transmission electron microscope (TEM) (HT7700, Hitachi, Tokyo, Japan) at 120 kV.

#### Contact angle tests

To evaluate the influence of HA on the hydrophilicity of PLLA fiber membrane, the contact angle of 2 μl water droplets on the surface of the sample was measured utilizing an optima goniometer (DSA25S, Zeiss, Oberkochen, Germany).

#### Measurement of mechanical properties

The mechanical characteristics of the nanofibrous membranes were evaluated using a mechanical testing apparatus (Instro-5542, Highwycombe, UK). Tensile assessments were conducted at a consistent velocity of 2.5 mm/min until the fibrous membrane experienced rupture.

#### Degradation rate

The dried nanofibrous membranes were initially weighed as *W*_0_ and subsequently submerged in 5 ml of phosphate-buffered saline (PBS). The samples were then incubated on a rocker at 37°C for 8 weeks. At predetermined intervals, the samples were rinsed with distilled water and weighed as *W_t_*. The degeneration rate was determined using the formula: Degradation rate (%) = *W_t_*/*W*_0_ × 100%.

#### Release profile of irisin

The irisin-loaded electrospun membranes were submerged in a 50-ml centrifuge tube containing 10 ml of PBS supplemented with 1% bovine serum albumin. The tube was then placed in a constant temperature shaker at 37°C. At predetermined intervals (1, 3, 5, 7, 9, 11, 14, 21, and 28 days), the supernatant was collected from the centrifuge tube and subsequently replaced with 10 ml of fresh PBS. The amount of irisin released from the electrospun membranes was quantified using the irisin enzyme-linked immunosorbent assay kit (R&D Systems, Minneapolis, MN, USA).

### *In vitro* experiments

#### Isolation and cell culture of rat BMMSCs

The isolation of rat BMMSCs was conducted using 8-week-old Sprague Dawley (SD) rats in accordance with a previously reported protocol [22]. Specifically, after sacrificing, the rat femurs and tibias were collected. The bone marrow was subsequently flushed out with alpha minimum essential medium (α-MEM, Thermo Fisher Scientific, Waltham, MA, USA), and the mononucleated cells were cultured in complete growth medium comprising α-MEM supplemented with 10% fetal bovine serum (FBS), 100 U/ml penicillin, and 100 μg/ml streptomycin (Thermo Fisher Scientific) at 37°C with 5% CO_2_. After 3 days, nonadherent cells were removed through medium change, and upon reaching confluence, the cells were trypsinized with 0.25% trypsin-ethylene diamine tetraacetic acid (EDTA) (Thermo Fisher Scientific) and reseeded. The cells at passage two (P2) were utilized for subsequent experiments.

#### Cell viability assay

The electrospun nanofibrous membranes underwent sterilization with 75% ethanol for 30 min, followed by an overnight immersion in α-MEM. Cell viability was determined using a live/dead staining kit (Beyotime, Haimen, China). Cells were seeded in 24-well plates at a density of 1 × 10^5^ cells per well. After 1, 3, and 5 days of culture, the cells were stained with the working solution of the live/dead cells staining for 30 min at 37°C. The stained cells were observed and captured using an automated fluorescence microscope (Zeiss).

#### Cell proliferation assay

Cell proliferation was quantified through the utilization of Cell Counting Kit-8 assays (CCK-8, Beyotime). Cells were seeded into 96-well plates at a density of 1 × 10^3^ cells per well and treated with the leachate from each group. At predetermined intervals (1, 3, 5, and 7 days), 100 μl of the CCK-8 solution was added and the cells were incubated for 1 h at 37°C. Optical density (OD) was subsequently measured at a wavelength of 450 nm using a multiplate reader (BioTek, Winooski, VT, USA).

#### Quantitative reverse transcription–polymerase chain reaction

The extraction of total RNA was performed using Trizol reagent (Thermo Fisher Scientific) and the subsequent synthesis of complementary DNA (cDNA) was carried out using the cDNA kit (Thermo Fisher Scientific). The reaction system for quantitative reverse transcription–polymerase chain reaction (RT-PCR) was prepared using the SYBR Green Master Supermix kit (Bio-Rad, CA, USA). The analysis of gene expression was conducted utilizing the CFX96TM real-time PCR instrument (Bio-Rad). Glyceraldehyde-3-phosphate dehydrogenase was determined as an internal control and the relative expression of target gene was calculated with *χ* = 2^-ΔΔ^^*CT*^ method. The primer sequences are listed in Supplementary Table S1.

#### Western blot

The protein was extracted using radioimmunoprecipitation assay buffer (Beyotime). The concentration of protein was determined using a BCA Protein Assay Kit (Beyotime). Subsequently, the protein samples were separated by 10% sodium dodecyl sulfate-polyacrylamide gel electrophoresis (Beyotime) and transferred onto a nitrocellulose membrane (Beyotime). Following blocking, the membranes were incubated with primary antibodies at 4°C overnight (Supplementary Table S2). The following day, the membranes were subsequently subjected to incubation with secondary antibodies labeled with horseradish peroxidase and were visualized through the use of an enhanced chemiluminescence solution (Thermo Fisher Scientific) and ChemiDoc Touch Imaging System (Bio-Rad). The resulting images were then subjected to semiquantitative evaluation through the utilization of ImageJ software (National Institutes of Health, Bethesda, MD, USA).

#### Mitochondrial membrane potential

The measurement of mitochondrial membrane potential (MMP) level was conducted using a commercially available JC-1 Assay Kit (Beyotime). The cells were subjected to incubation with JC-1 working solution at 37°C for 20 min, as per the manufacturer’s instructions. The visualization of mitochondria was accomplished using an Axiovert 40CFL microscope (Zeiss). Red fluorescence was observed from JC-1 aggregates, while green fluorescence was observed from JC-1 monomers. The calculation of MMP level was based on the ratio of red to green fluorescence intensity.

#### Immunofluorescence staining

BMMSCs in 24-well plates were fixed with 4% paraformaldehyde (Aladdin, Shanghai, China), followed by permeabilization with 0.1% Triton X-100 (Beyotime) for 10 min. After blocking for 30 min, the cells were incubated with antibodies at 4°C for 8 h. Subsequently, the cells were incubated with a secondary antibody (ab150075, Abcam, Cambridge, UK) conjugated with phalloidin (Beyotime). Finally, the nucleus was counterstained with DAPI (Beyotime), and images were captured using a fluorescence microscope (Zeiss).

#### Measurement of intracellular and mitochondrial ROS

To assess intracellular ROS, the cells were incubated with 5 μM 2′,7′-dichlorodihydrofluorescein diacetate (DCFH-DA, Beyotime) at 37°C for 20 min. Mitochondrial ROS levels were evaluated by incubating the cells with 5 μM MitoSOX^TM^ red (Beyotime). Then the images were captured using a fluorescence microscope (Zeiss).

#### Measurement of adenosine triphosphate production

BMMSCs cultured on the membranes were subjected to lysis and, after centrifugation, the supernatant was treated with the adenosine triphosphate (ATP) detection solution at 4°C for 5 min (Beyotime). The OD of each well was measured using a Centro LB960 instrument (Berthold Technologies, Bad Wildbad, Germany). To standardize the ATP levels, the protein concentration of each sample was determined using a bicinchoninic (BCA) acid protein assay kit (Beyotime).

### *In vitro* osteogenic induction

BMMSCs were seeded on nanofibrous membranes and cultured with α-MEM medium containing 10% FBS, 10 mM β-glycerol phosphate, 100 nM dexamethasone, and 50 μg/ml l-ascorbic acid (all from Sigma-Aldrich) for 21 days. The osteogenic medium was replenished every 3 days. On the seventh day, alkaline phosphatase (ALP) activity was determined using an ALP staining kit (Beyotime) and an ALP quantification kit (Jiancheng, Nanjing, China). Matrix mineralization was observed using an Alizarin Red Staining (ARS) kit (Cyagen, Guangzhou, China). Following the differentiation period, the cells were fixed in 4% paraformaldehyde and treated with 40 mM ARS solution at room temperature for 30 min. Digital images were captured using an Olympus IX51 microscope (Olympus, Tokyo, Japan). Quantitative analysis was performed by adding 5% perchloric acid solution to each well and measuring the absorbance at a wavelength of 420 nm using a spectrophotometer (Bio Tek).

### *In vivo* experiments

Eighteen male SD rats, with an average weight of 300–350 g, were procured from the Experimental Animal Center of Soochow University. The surgical interventions were carried out in compliance with the guidelines endorsed by the Ethics Committee of Soochow University (SUDA20221228A02). The rats were housed under controlled conditions, including constant humidity (50–60%), temperature (22–24°C), and a light cycle from 6 am to 6 pm.

#### Establishment of a rat critical size calvarial defect model

The induction of anesthesia in SD rats was accomplished through the intraperitoneal administration of 2% pentobarbital sodium (40 mg/kg, Chemical Reagent Company, Shanghai, China). Following complete shaving and disinfection, a longitudinal incision was made in the center of the surgical site, and the soft tissue was meticulously separated to expose the calvarium. After the periosteum was removed, two full-thickness defects (4 mm in diameter) were carefully created by an electric drill on the calvarium and subsequently covered with different groups of electrospun nanofibrous membranes. The outer diameter of the electric drill bit is 4 mm, the inner diameter is 3 mm and the thickness is 1 mm. The experimental groups were categorized into three groups, namely the defect, PLLA/HA and PLLA/HA@Irisin groups. It is noteworthy that the treatment employed for skull defects in the PLLA/HA@Irisin group involved the utilization of the PLLA/HA@Irisin(H) membrane. Postoperatively, penicillin was administered once daily for three consecutive days.

#### Micro-computed tomography scanning

Following a period of 4 and 8 weeks of implantation, the calvarial specimens were harvested and fixed in 10% formalin (Jiancheng) for further analysis. The defect areas were assessed using micro-computed tomography (CT) (SkyScan 1176, Bruker Corporation, Billerica, MA, USA). The three-dimensional (3D) structures of the calvarium were reconstructed using Mimics software (Materialise, Leuven, Belgium). A cylindrical space was designated to represent the region of interest to calculate bone volume/tissue volume (BV/TV, %) and trabecular thickness (Tb.Th, μm) through CT Analyzer software (Bruker).

#### Histological analysis

The calvarial samples underwent fixation and decalcification using a 10% EDTA for 6 weeks at room temperature. Subsequently, the specimens were subjected to alcohol gradient dehydration and embedded in a paraffin block. The embedded samples were then sectioned into 5 µm-thick sections, which were positioned across the center of the defect area.

Hematoxylin-eosin (H&E) staining was performed by incubating the sections in hematoxylin (Solaibao Technology Co., Ltd, Beijing, China) for 2 min and eosin (Solaibao) for 1 min, respectively. Masson’s trichrome staining (MTS, Solaibao) was conducted according to the manufacturer’s protocol to assess the collagen deposition. The sections were subjected to dewaxing and hydration, followed by staining with a hematoxylin and lichun acid solution for 5 min each. Subsequently, the sections were immersed in a weak acid solution for a brief period, differentiated with phosphomolybdic acid solution for 1 min, and finally stained with aniline blue for 1 min. The images were captured utilizing a bright field microscope (Zeiss).

#### Immunohistochemical staining

The sections underwent deparaffinization using xylene, hydration with graded ethanol and treatment with 3% hydrogen peroxide (Sigma-Aldrich) to inhibit endogenous peroxidase. Subsequently, the sections were incubated with 2 mg/ml of testicular hyaluronidase (Sigma-Aldrich) for 30 min at 37°C, blocked with 1.5% goat serum for 30 min, and finally incubated with specific anti-CD31 (1:500, A0378, ABclonal, Wuhan, China) or anti-COL I (1:500, ab138492, Abcam) primary antibodies overnight at 4°C. On the next day, the tissues underwent additional incubation with biotinylated goat anti-rabbit secondary antibody (Vector Laboratories, Burlingame, CA, USA) for 30 min. Subsequently, signal amplification was executed using the vecastain Elite ABC kit (Vector Laboratories). Staining was accomplished using 3,3′-diaminobenzidine solution (DAB, Vector Laboratories), while nuclear counterstaining was achieved using hematoxylin. Bright-field microscopy was utilized to capture images, and the quantification of positive cells was performed using ImageJ software.

### Statistical analysis

Data were acquired from a minimum of three independent experiments and presented as the means ± standard error of the mean (SEM). The double-tailed Student’s *t*-test was utilized to compare two groups, while one-way analysis of variance with Tukey’s *post hoc* test was employed for multiple group comparisons. Statistical significance was denoted by * (*P *<* *0.05) or ** (*P *<* *0.01) between the specified groups. All statistical analyses were conducted using SPSS 13.0 statistical software (SPSS Inc., Chicago, IL, USA).

## Results

### Characterization of PLLA/HA@Irisin nanofibrous membranes

**Figure 2.**
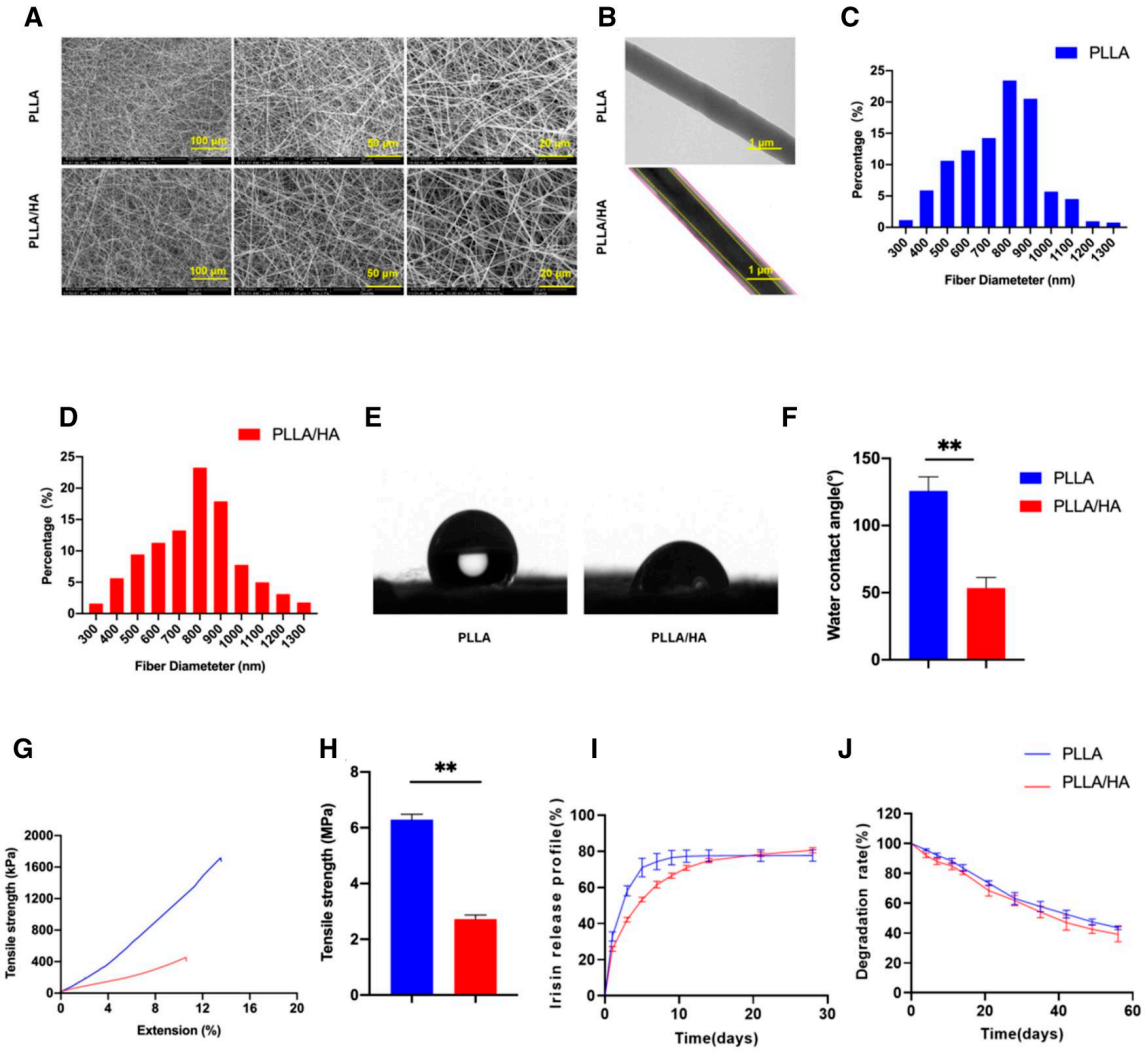
Morphology and characterizations of different membranes. (**A**) Representative images of SEM. (**B**) Representative images of TEM. (**C**) The pore size distribution of the PLLA membranes. (**D**) The pore size distribution of the PLLA/HA membranes. (**E**) The images of water contact angles of PLLA and PLLA/HA membranes. (**F**) The quantification of water contact angles, *n* = 3. (**G**, **H**) The stress-strain curve and the tensile strength of the PLLA and PLLA/HA membranes, *n* = 3. (**I**) Irisin release profile at each time point within 30 days, *n* = 3. (**J**) Degradation rate of PLLA and PLLA/HA membranes within 8 weeks, *n* = 3. Statistically significant differences were indicated by *P <* *0.05 or **P <* *0.01.

SEM results demonstrated that the electrospun nanofibers displayed a disordered configuration, leading to the creation of numerous pores. The resemblance of the microstructure to the native ECM proved advantageous in facilitating cell adhesion, migration and proliferation (Figure 2A). TEM analysis further revealed that the PLLA/HA nanofibers possessed a uniform core-shell structure, which differed significantly from the structure of PLLA nanofibers (Figure 2B). The average diameters of both PLLA and PLLA/HA fibers fell within the range of 790.0 ± 196.4 and 781.6 ± 191.0 nm (Figure 2C and D). The water contact angles of the PLLA and PLLA/HA membranes were measured as 125.8 ± 10.5° and 53.0 ± 7.6°, respectively (Figure 2E and F). The incorporation of HA in the outer layer of the spinning film led to a significant decrease in hydrophilicity angle, owing to the superior hydrophilicity of HA. Additionally, an assessment of the mechanical properties of the two membrane types was conducted. The tensile modulus of the PLLA membrane was determined to be 6.3 ± 0.2 MPa, whereas the PLLA/HA membrane exhibited a reduced value of 2.7 ± 0.2 MPa (Figure 2G and H), indicating that the addition of HA resulted in a decrease in the mechanical strength of the PLLA nanofibers (Figure 2G and H). However, the release profile demonstrated that the core-shell structure of PLLA/HA nanofibers successfully achieved sustained release of irisin. After a period of 10 days, the irisin released from the PLLA membrane was barely detectable. In contrast, the PLLA/HA membrane exhibited continuous release of irisin for a duration of 30 days, indicating a prolonged drug release behavior (Figure 2I). Upon immersion in PBS for 8 weeks *in vitro*, the degradation rates of the PLLA and PLLA/HA membranes were measured at 51.5% and 43.8%, respectively (Figure 2J).

### Biocompatibility of PLLA/HA@Irisin nanofibrous membranes

**Figure 3.**
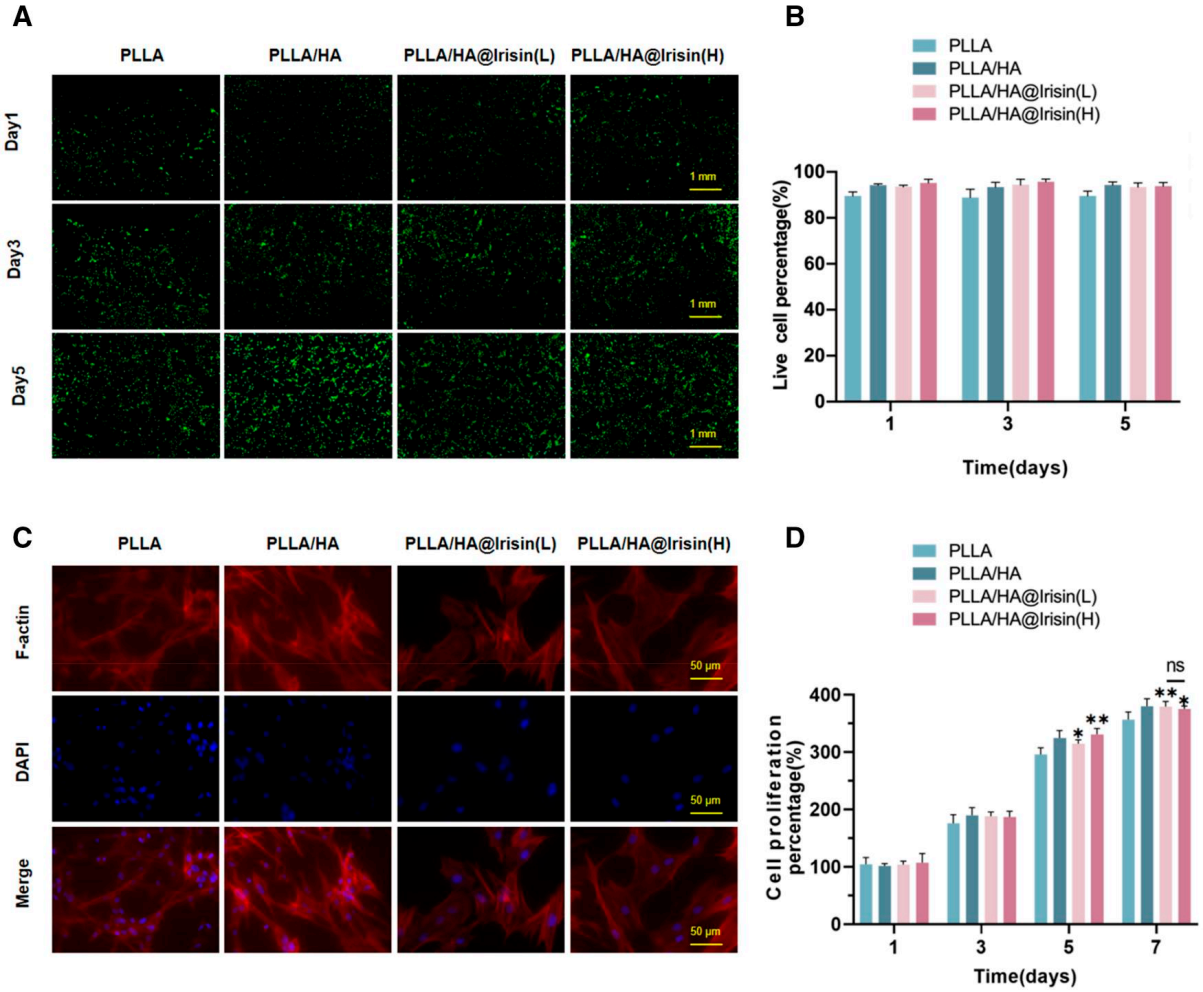
The biocompatibility of PLLA/HA membranes loaded with irisin. (**A**) The cell viability of BMMSCs cultured on electrospun nanofibers was determined using the live/dead cell staining. Scale bar = 1 mm. (**B**) Quantification of the live cells cultured on electrospun nanofibers, *n* = 3. (**C**) The cytoskeleton and nuclear staining of BMMSCs cultured on the nanofibrous membranes. Scale bar = 50 μm. (**D**) Cell proliferation rate was determined by CCK-8 assays, *n* = 4. Statistically significant differences were indicated by *P <* *0.05.

BMMSCs were cultured on four different types of membranes for a duration of 5 days. The live/dead cell staining analysis revealed that the presence of PLLA/HA membranes had minimal impact on cell viability compared to the PLLA group. Furthermore, the PLLA/HA@Irisin(L) and PLLA/HA@Irisin(H) membranes exhibited no noticeable cytotoxic effects on BMMSCs (Figure 3A and B). Cytoskeleton staining demonstrated that these irisin-loaded membranes promoted cell spreading (Figure 3C). Specifically, when compared to the PLLA group, the spread area was observed to increase by 47.5% and 65.1% for PLLA/HA@Irisin(L) and PLLA/HA@Irisin(H), respectively (Supplementary Figure S1). Additionally, the proliferation of BMMSCs in the presence of irisin-loaded PLLA/HA membranes was assessed using CCK-8 assays. In comparison to the PLLA group, the PLLA/HA@Irisin(L) and PLLA/HA@Irisin(H) membranes exhibited a statistically significant enhancement in the cell proliferation rate of BMMSCs (Figure 3D). These findings indicated that the irisin-loaded PLLA/HA membranes, featuring a core-shell structure, possess favorable biocompatibility.

### PLLA/HA@Irisin membranes promoted the osteogenic differentiation of BMMSCs

**Figure 4.**
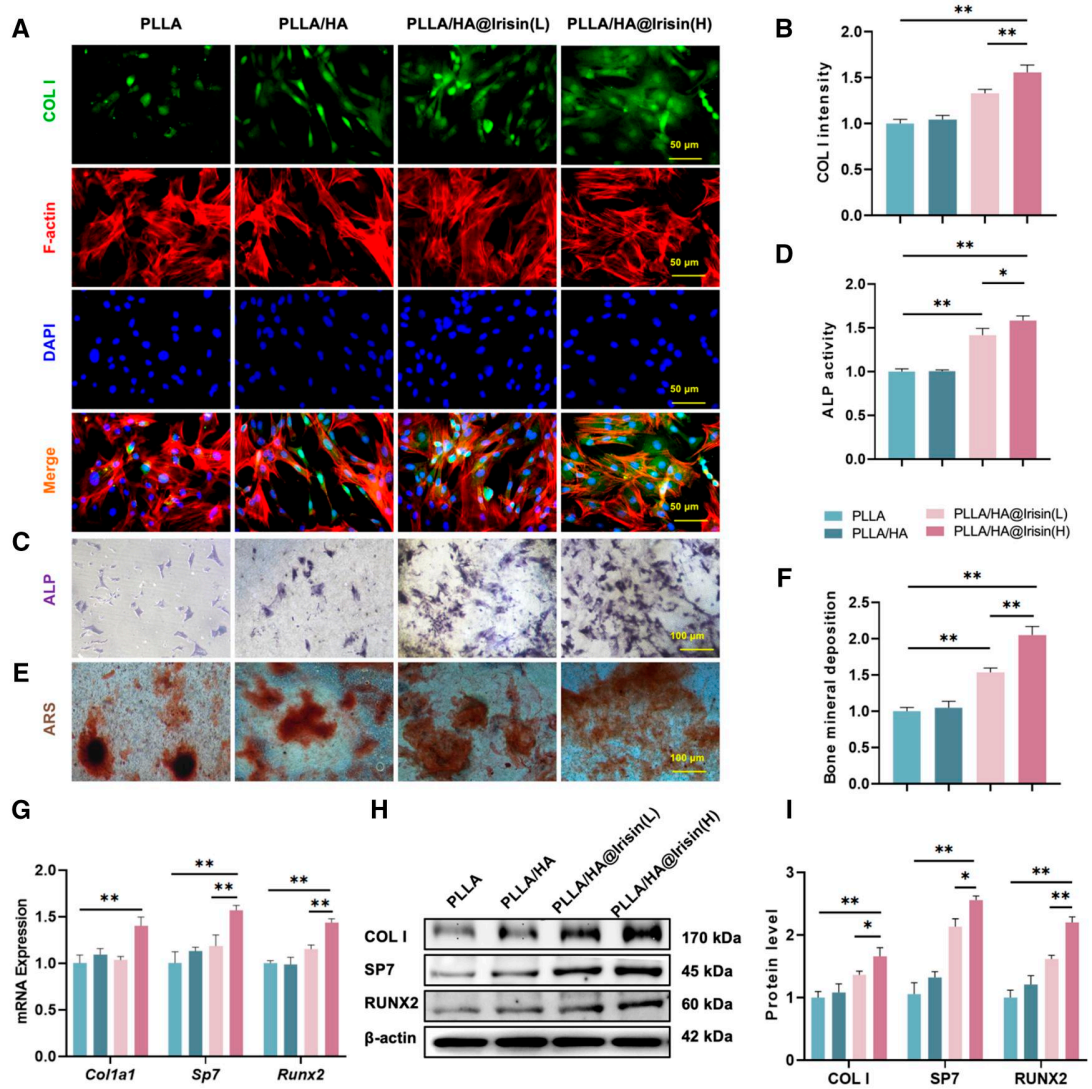
PLLA/HA@Irisin membranes promoted the *in vitro* osteogenic differentiation of BMMSCs. (**A**, **B**) The expression level of COL I was evaluated by immunofluorescence staining, *n* = 3. Scale bar = 50 μm. (**C**) After 7 days of osteogenic induction, ALP staining was performed on BMMSCs scale bar = 100 μm. (**D**) Quantitative results of ALP activity of BMMSCs, *n* = 3. (**E**) Representative images of bone mineral deposition in BMMSCs stained by ARS. Scale bar = 100 μm. (**F**) Quantification of the stained bone mineral deposition in BMMSCs cultured on different groups of membranes, *n* = 3. (**G**) The gene expression of osteogenic makers, including *Col1a1*, *Sp7*, and *Runx2*, was determined by RT-PCR, *n* = 4. (**H**, **I**) The protein levels of COL I, SP7 and RUNX2 were examined by western blot, *n* = 3. Statistically significant differences were indicated by *P <* *0.05 or **P <* *0.01.

BMMSCs were cultivated on various nanofibrous membranes and stimulated toward osteogenic differentiation. Immunofluorescence staining revealed a noteworthy augmentation of COL I in BMMSCs cultured on irisin-loaded PLLA/HA membranes (Figure 4A). The results of the quantification analysis demonstrated that the PLLA/HA@Irisin(L) and PLLA/HA@Irisin(H) formulations exhibited a significant up-regulation in the protein expression of COL I, with increases of 33.0% and 55.8%, respectively, when compared to the PLLA control group (Figure 4B). Furthermore, the ALP activity, a well-established marker for early-stage osteogenic differentiation, was found to be 41.5% and 58.2% higher in BMMSCs cultured on PLLA/HA@Irisin(L) and PLLA/HA@Irisin(H), respectively, after a 7-day period of osteogenic induction, in comparison to the PLLA group (Figure 4C and D). After a 21-day of osteogenic induction, the application of ARS staining revealed that the calcium deposition on the PLLA/HA@Irisin(L) and PLLA/HA@Irisin(H) membranes exhibited a significant improvement of 53.5% and 105.1%, respectively, when compared to the PLLA group (Figure 4E and F). Additionally, the expression of osteogenic markers in BMMSCs cultured on PLLA/HA@Irisin nanofibers was found to be up-regulated. Notably, PLLA/HA@Irisin(H) demonstrated a significant increase in the transcript levels of *Col1a1* by 38.8%, *Sp7* by 74.7% and *Runx2* by 89.7% in comparison to the PLLA group (Figure 4G). The results of western blot demonstrated a significant up-regulation of protein expression levels of COL I, SP7 and RUNX2 by 65.8%, 150.1% and 120.1%, respectively, in the PLLA/HA@Irisin(H) group, as compared to the PLLA group (Figure 3H and I). These findings provide compelling evidence that the utilization of irisin-loaded PLLA/HA membranes effectively enhances the *in vitro* osteogenic differentiation of BMMSCs.

### PLLA/HA@Irisin nanofibrous membranes improved the mitochondrial energy metabolism of BMMSCs

**Figure 5.**
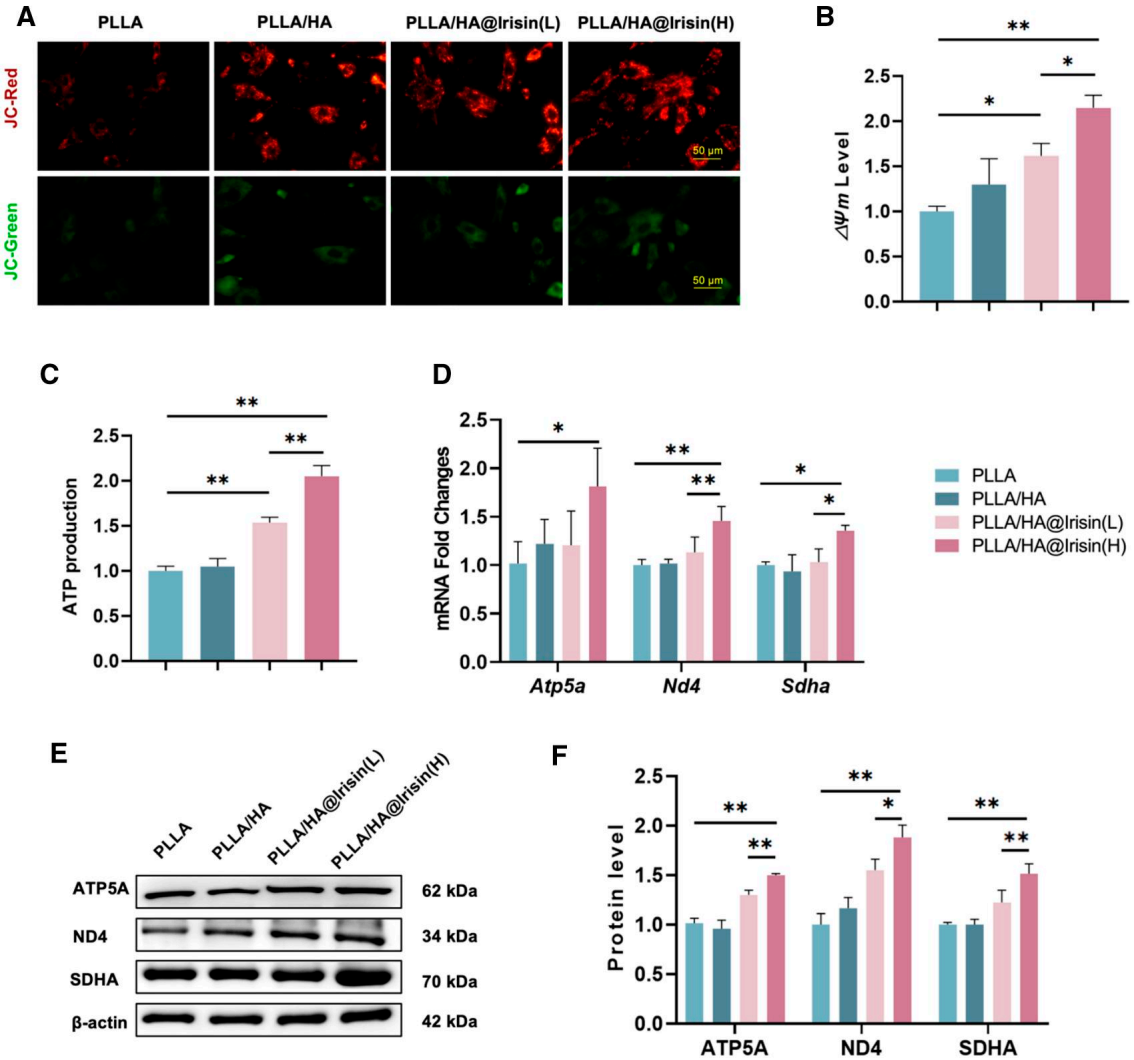
PLLA/HA@Irisin membranes improved the mitochondrial function of BMMSCs. (**A**, **B**) JC-1 staining indicated the MMP of BMMSCs, *n* = 3. Scale bar = 5μm. (**C**) ATP production of BMMSCs, *n* = 4. (**D**) The gene expression of mitochondrial respiratory chain factors, including *Atp5a*, *Nd4* and *Sdha*, was determined by quantitative RT-PCR, *n* = 4. (**E**, **F**) The protein levels of ATP5A, ND4 and SDHA were examined by western blot, *n* = 3. Statistically significant differences were indicated by *P <* *0.05 or **P <* *0.01.

The influence of PLLA/HA@Irisin nanofibers on the mitochondrial function of BMMSCs was evaluated in the context of bone regeneration. JC-1 staining was utilized to evaluate the mitochondrial permeability of BMMSCs, which serves as a crucial indicator for assessing mitochondrial integrity (Figure 5A). The levels of MMP in BMMSCs cultured on PLLA/HA@Irisin(L) and PLLA/HA@Irisin(H) were observed to exhibit a respective increase of 61.6% and 114.6%, respectively, compared to the PLLA group (Figure 5B). This consistent finding suggests that PLLA/HA@Irisin effectively enhances mitochondrial energy metabolism. Furthermore, the production of ATP, a key indicator of mitochondrial energy metabolism, was significantly elevated by 17.0% with PLLA/HA@Irisin(L) and by 36.2% with PLLA/HA@Irisin(H) in comparison to the PLLA group (Figure 5C). Additionally, the impact of PLLA/HA@Irisin membranes on the expression of genes related to the mitochondrial respiratory chain was investigated. The PLLA/HA@Irisin(H) treatment demonstrated a significant enhancement in the gene expression of *Atp5a* (ATP synthase F1 subunit alpha), *Nd4* (NADH dehydrogenase subunit 4), *Sdha* (succinate dehydrogenase complex flavoprotein subunit A), by 79.7%, 30.6% and 45.5% respectively, compared to the PLLA group (Figure 5D). This finding was further supported by western blot analyses, which indicated a similar trend in the protein levels of ATP5A, ND4 and SDHA. The PLLA/HA@Irisin(H) group exhibited an increase of 48.5%, 88.0% and 43.0%, respectively, compared to the PLLA group (Figure 5E and F). These data demonstrated that the use of PLLA/HA@Irisin resulted in an increase in energy production and an improvement in mitochondrial function in BMMSCs. To ascertain potential disparities in the osteogenic properties of irisin released by the PLLA/HA@Irisin(H) membranes and the normal irisin recombinant protein, BMMSCs were exposed to the extract of the membranes (day 1) and an equivalent concentration (250 ng/ml) of irisin solution. The findings revealed no statistically significant distinction in their ability to promote osteogenesis (Supplementary Figure S2).

### PLLA/HA@Irisin enhanced the mitochondrial function of BMMSCs by activating the SIRT3 pathway

**Figure 6.**
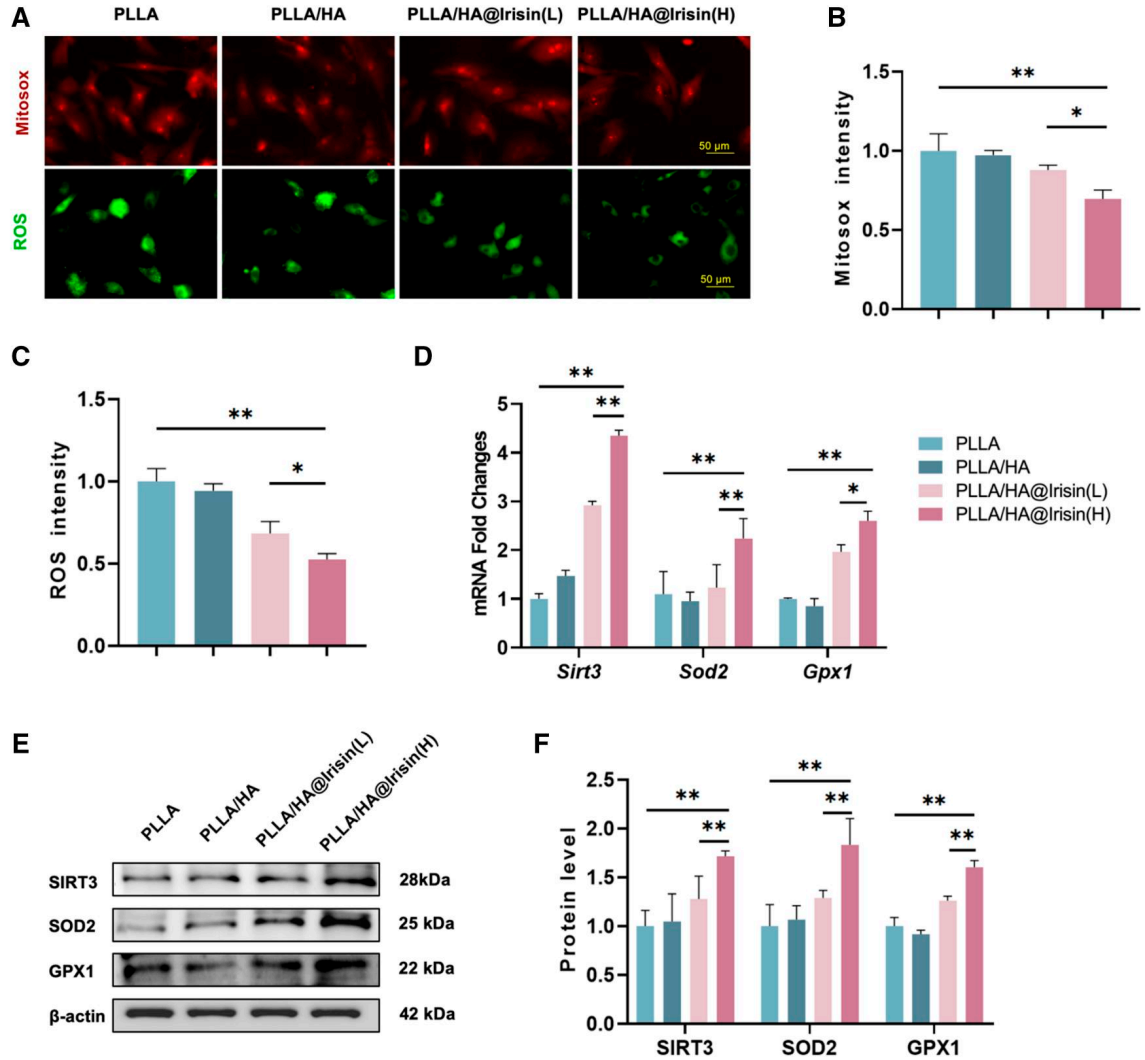
PLLA/HA@Irisin membranes enhanced the antioxidant functions of BMMSCs. (**A**) Mitochondrial and intracellular ROS were detected through MitoSOX and DCFH-DA staining, respectively. Scale bar = 50 μm. (**B**) Quantification of mitochondrial ROS in BMMSCs, *n* = 3. (**C**) Quantification of intracellular ROS in BMMSCs, *n* = 3. (**D**) The gene expression of *Sirt3*, *Sod2* and *Gpx1* was determined by RT-PCR. (**E**, **F**) Western blot was used to determine the protein levels of SIRT3, SOD2 and GPX1. Statistically significant differences were indicated by *P <* *0.05 or **P <* *0.01.

Considering the importance of redox homeostasis in the process of bone regeneration, we conducted further investigations to examine the impact of PLLA/HA@Irisin on the production of ROS and the protection against oxidative stress. To evaluate the levels of mitochondrial and intracellular ROS, immunofluorescence staining was employed (Figure 6A). The results revealed a significant reduction of 30.4% and 47.4% in mitochondrial (Figure 6B) and intracellular (Figure 6C) ROS levels, respectively, in BMMSCs cultured on PLLA/HA@Irisin(H) compared to the PLLA group. Moreover, PLLA/HA@Irisin demonstrated a significant enhancement in the up-regulation of intracellular antioxidant enzymes in BMMSCs, as evidenced by both mRNA and protein levels. Particularly, PLLA/HA@Irisin(H) exhibited a remarkable increase in the gene expression of *Sirt3* by 3.5-fold, *Sod2* by 1.0-fold and *Gpx1* by 1.6-fold compared to the PLLA group (Figure 6D). Furthermore, western blot assays revealed that the expression of antioxidant-related proteins in BMMSCs cultured on PLLA/HA@Irisin(H) membranes was elevated by 71.6% for SIRT3, 83.2% for SOD2 and 60.2% for GPX1, in comparison to the PLLA group (Figure 6E and F). According to these findings, SIRT3-mediated intracellular antioxidant enzymes were activated by PLLA/HA@Irisin to regulate the redox homeostasis in BMMSCs.

### Implantation of PLLA/HA@Irisin membranes boosted vascularized bone regeneration in critical-sized calvarial defects

**Figure 7.**
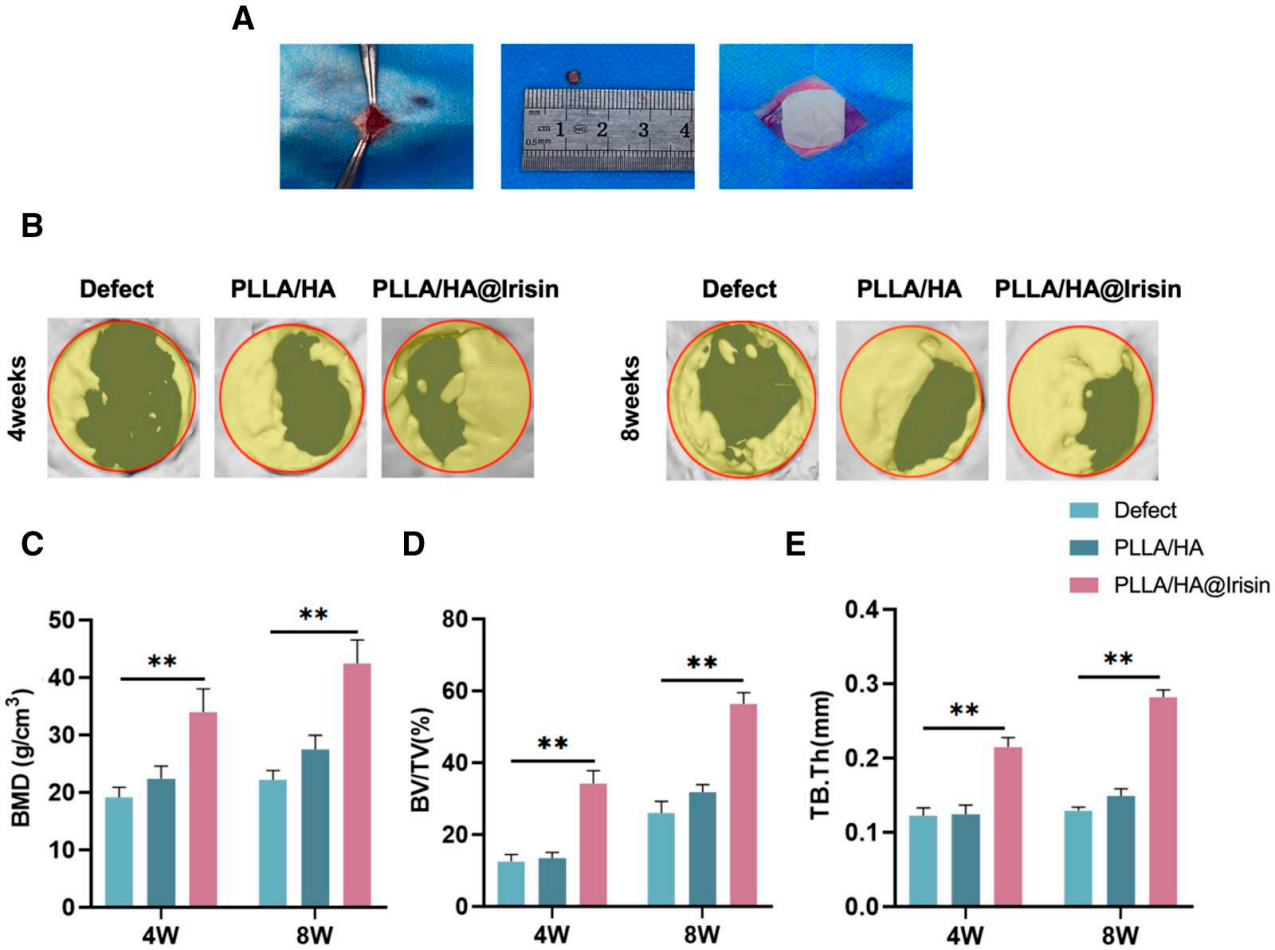
Micro-CT evaluation of *in vivo* bone regeneration of critical-sized calvarial defects. Two full-thickness defects (4 mm in diameter) were drilled on rat calvarium. PLLA/HA and PLLA/HA@Irisin nanofibrous membranes were *in situ* implanted. The untreated defects served as the defect group. (**A**) Schematic diagram of surgery and implantation process. (**B**) At 4 and 8 weeks post-surgery, the new bone formation in the calvarial defect was evaluated by micro-CT imaging and 3D reconstruction. Quantitative analysis of (**C**) BMD, (**D**) bone volume ratio (BV/TV) and (**E**) Tb.Th in the defect area after 4 and 8 weeks of implantation, *n* = 3. Statistically significant differences were indicated by *P <* *0.05 or **P <* *0.01.

To assess the therapeutic efficacy of PLLA/HA@Irisin nanofibrous membranes, two full-thickness defects of the rat calvarium were carefully created and covered with PLLA/HA and PLLA/HA@Irisin membranes (Figure 7A). The application of micro-CT imaging demonstrated that the implementation of PLLA/HA@Irisin effectively facilitated bone regeneration at 4 and 8 weeks following surgery, whereas the defect group exhibited insufficient healing (Figure 7B). The quantitative analysis revealed that the implementation of PLLA/HA@Irisin resulted in a notable increase in bone mineral density (BMD), with levels that were 77.4% higher at 4 weeks post-surgery and 91.2% higher at 8 weeks post-surgery compared to the defect group (Figure 7C). Furthermore, at 8 weeks post-surgery, the PLLA/HA@Irisin group exhibited a significant enhancement in BV/TV and Tb.Th, with values that were 116.6% (Figure 7D) and 117.8% (Figure 7E) higher, respectively, in comparison to the defect group.

**Figure 8.**
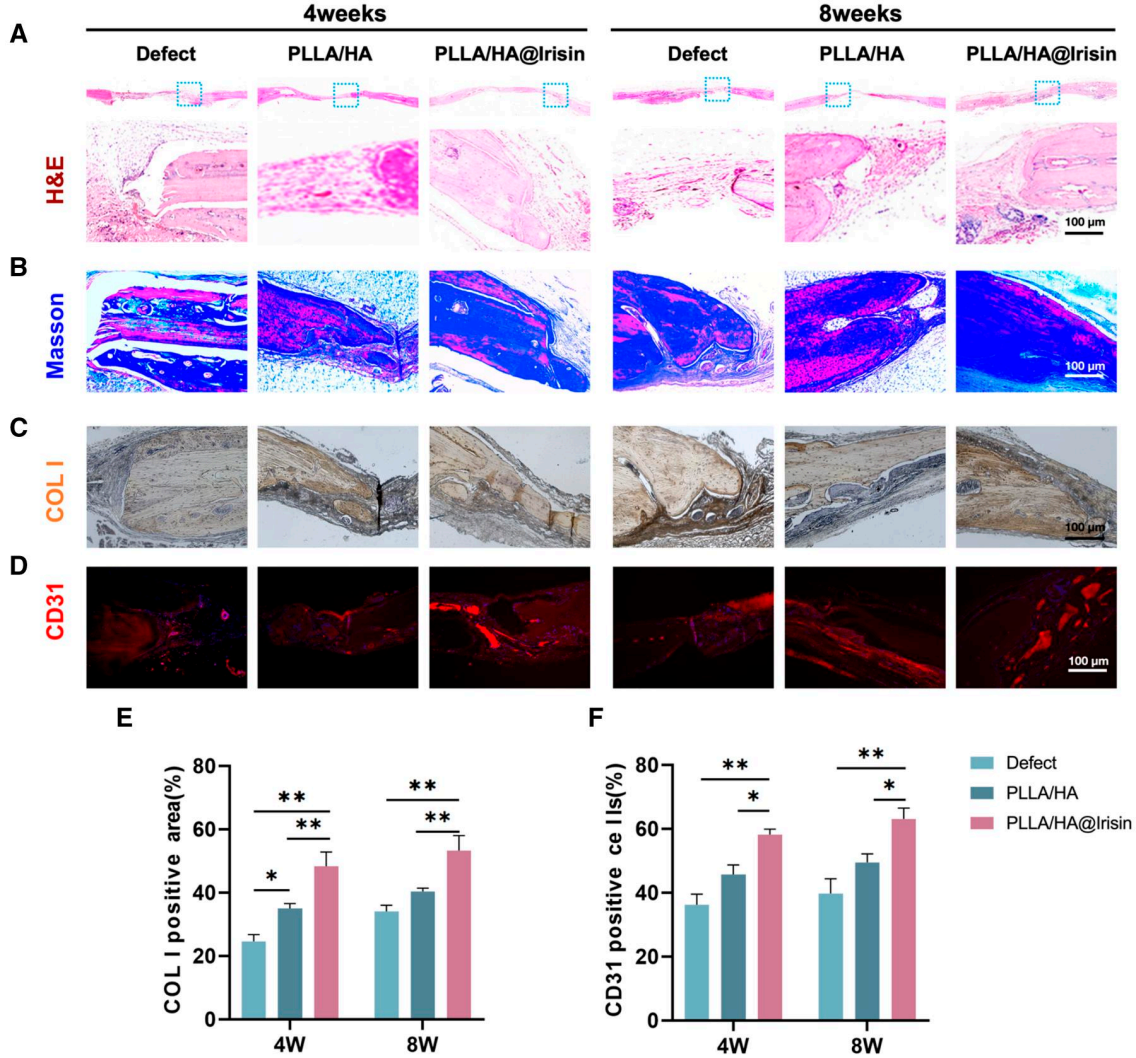
Histological and immunohistochemical analysis of the newly formed bone tissue in the calvarial defects. (**A**) H&E images of the defect area at 4 and 8 weeks post-surgery. (**B**) MTS images of the defect area at 4 and 8 weeks post-surgery. (**C**) IHC images of COL I in the defect area. (**D**) IHC images of CD31-positive cells that represented the newly formed blood vessels. Scale bar = 100 μm. (**E**) Quantification of the COL I-positive area, *n* = 3. (**F**) Quantification of the CD31-positive cells, *n* = 3. Statistically significant differences are indicated by *P <* *0.05 or **P <* *0.01.

Histological analysis was performed to assess the bone regeneration of the defect area. At 4 and 8 weeks after the surgical procedure, the application of PLLA/HA@Irisin nanofibrous membranes was found to promote the healing of calvarial defects, as indicated by the presence of numerous bone formation islands exhibiting densely packed trabecular patterns, as observed through H&E staining (Figure 8A). MTS was utilized to investigate collagen deposition at the defect site, revealing a substantial amount of positive collagen staining in the PLLA/HA@Irisin group, indicating the formation of new bone tissue (Figure 8B). Additionally, IHC provided confirmation of a significant positive staining for COL I and CD31 (Figure 8C and D). Comparative analysis revealed that the expression level of COL I in the defect area exhibited a 2.7-fold increase at 4 weeks post-surgery and a 1.5-fold increase at 8 weeks post-surgery subsequent to the implantation of PLLA/HA@Irisin (Figure 8E). Furthermore, the percentages of CD31-positive cells in the PLLA/HA@Irisin group were observed to be 100.0% higher at 4 weeks post-surgery and 88.1% higher at 8 weeks post-surgery compared to the defect group (Figure 8F). These findings serve as evidence that the implantation of PLLA/HA@Irisin membranes effectively enhances bone regeneration and facilitates the formation of new blood vessels in rat calvarial defects.

## Discussion

The analogous functions of irisin in osteogenesis have been reported successively, which is a polypeptide hormone secreted by skeletal muscle [23]. Both exogenous administration [24] and endogenous secretion [25] of irisin have been shown to prevent the apoptosis of osteoblasts, thereby augmenting cortical bone thickness and mineralization. Irisin is a variant resulting from the proteolytic cleavage of the fibronectin-type III domain-containing protein 5 (FNDC5). Global deletion of *Fndc5* gene (*Fndc5*^−/−^) in mice resulted in a dramatic decrease in bone mass compared to wild-type animals [24]. The exogenous treatment with irisin restored the osteogenic potential of MSCs derived from *Fndc5*^−/−^ mice by activating the extracellular regulated protein kinases and signal transducer and activator of transcription signaling pathways, which in turn activated the downstream osteogenic promoters, such as bone morphogenetic protein 2 (BMP2) and drosophila mothers against decapentaplegic protein [24]. Consistent with these results, the conditional knockout of *Fndc5* in bone tissue in mice caused significant growth retardation and delayed matrix mineralization of during the embryonic development [26]. Furthermore, the promotion of osteogenic differentiation by irisin is primarily attributed to the activation of the wingless-type MMTV integration site family (Wnt)/β-catenin signaling pathway, which plays a crucial role in bone formation by regulating the differentiation of MSCs into osteoblasts, as well as promoting bone matrix synthesis and calcium deposition [23]. Irisin has also been discovered to facilitate osteogenic differentiation through the regulation of crucial genes implicated in bone formation. Specifically, irisin has been demonstrated to elevate the expression of RUNX2 and SP7, the critical transcription factors that play an indispensable role in osteoblast differentiation [27]. Consequently, our investigation revealed that irisin released from the PLLA/HA@Irisin membranes effectively promoted osteogenic differentiation of BMMSCs *in vitro*, as indicated by the heightened expression of osteogenic markers and the augmented mineralization of bone matrix.

Previous studies have suggested that irisin played a crucial role in the regulation of mitochondrial function and energy metabolism. Irisin has been shown to enhance the expression of genes associated with mitochondrial biogenesis, such as PGC-1α and nuclear respiratory factor 1 (NRF-1) [28], both of which are transcription factors that facilitate the expression of genes involved in the biological functions of mitochondria [29]. The inner mitochondrial membrane houses the respiratory chain, which facilitates the ATP generation via oxidative phosphorylation [30]. This chain comprises four protein complexes (I–IV) and two electron carriers (ubiquinone and cytochrome c), which collaborate to transport electrons from nicotinamide adenine dinucleotide (NADH) and flavin adenine dinucleotide hydrogen transmitter 2 (FADH2) to oxygen, thereby producing a proton gradient that drives ATP synthesis [31]. The findings of our study suggested that irisin played a role in the stabilization of MMP and the activation of subunits within mitochondrial respiratory chain complexes, specifically SDHA, ND4, and ATP5A. Additionally, irisin has been observed to enhance the activity of complex I and complex IV within the respiratory chain, as well as increase the rate of electron transport and oxygen consumption in isolated mitochondria. These beneficial effects on mitochondrial function are facilitated possibly through the activation of AMP-activated protein kinase (AMPK), a crucial regulator of cellular energy metabolism [32]. AMPK serves as a cellular energy sensor that is triggered by alterations in the AMP/ATP ratio. Its activation results in heightened mitochondrial biogenesis and oxidative capacity, while simultaneously reducing lipid synthesis and adiposity [33]. Irisin has been demonstrated to activate AMPK in both skeletal muscle cells and adipose tissue, thereby promoting mitochondrial function and energy expenditure [34].

Oxidative stress is characterized by an imbalance between ROS production and the antioxidant defense system, resulting in cellular damage and dysfunction [35]. Irisin has been identified as a potential protective agent against oxidative stress-induced apoptosis in an experimental study. Exposure cardiomyocytes to hydrogen peroxide caused serious oxidative damage to cells, whereas irisin treatment effectively protected the cell viability through attenuation of ROS levels [36]. Additionally, irisin is able to increase the expression of antioxidant enzymes, including SOD and catalase, which further mitigated the effects of oxidative stress [37]. Another study investigated the effects of irisin on oxidative stress-induced cell death, suggesting that the protective effect of irisin was through up-regulation of the expression of antioxidant enzymes including SOD, GPx and HO-1 [38]. Our study discovered that irisin activated SOD1 and GPx1, resulting in decreased levels of ROS in BMMSCs and protection of the mitochondrial from oxidative injury. Additionally, irisin was found to up-regulate the expression of SIRT3, an upstream factor that regulates a variety of antioxidant enzymes [39]. Consistent with our findings, irisin has been documented to provide protection against SIRT3 down-regulation caused by oxidative stress [40]. It has been proposed that the up-regulation of SIRT3 by irisin possibly involves the activation of the AMPK and PGC-1α signaling pathways [41]. PGC-1α is a transcriptional coactivator that plays a role in the regulation of mitochondrial biogenesis and oxidative homeostasis [42]. It is noteworthy that irisin activates antioxidant signaling pathways, which also contributes to the enhancement of mitochondrial stability.

Prior researches have utilized intraperitoneal injection of irisin as a means of promoting bone healing [43, 44]. However, achieving a sufficient concentration of irisin at the site of injury via intraperitoneal injection proved challenging. In light of this, we developed a nanofibrous membrane with a core-shell structure composed of PLLA and HA, which enabled the loading of irisin. The inner layer of the membrane facilitated sustained drug release for up to 1 month post-implantation, thereby satisfying the requirements for bone regeneration. The membrane exhibits potential as a viable approach for the regeneration of periosteal tissue, owing to its mechanical characteristics and barrier functions [45]. Synthetic polymer-based spinning membranes, including polycaprolactone (PCL), poly(lactic-co-glycolic acid) (PLGA) and polyethylene glycol (PEG), are thin, flexible and biocompatible [46]. These membranes can be readily produced using a range of spinning techniques, including electrospinning, solution spinning and melt spinning [47]. In previous studies, it was identified that some bioactive factors, including bFGF [48], VEGF [49] and TGFβ [50] were carried on the inner layer of nanofibers, their outer layer of fibers is composed of PLLA, an almost nondegradable artificial polymer. However, the drug release curve indicated that the active factors were released over a period of approximately 4 weeks, aligning closely with the results of irisin release curve in our study. Furthermore, apart from PLLA, other widely employed nondegradable synthetic polymers are utilized as outer layer materials. Zhu *et al.* similarly integrated bioactive factors within the inner layer of fibers, employing a nearly nondegradable PCL as the outer shell. The drug exhibited a release duration of approximately 24 days, thereby failing to showcase the potential benefits associated with the utilization of nondegradable materials as the outer shell [51]. Another significant rationale for employing HA as an external coating lies in its exceptional hydrophilicity. It is widely acknowledged that micron-scale roughness and wettability significantly impact cell diffusion and differentiation [52]. A heightened hydrophilic characteristic of a biomaterial surface can augment cell growth and foster biocompatibility. By furnishing a more hydrophilic surface for spinning membranes, HA facilitates a more conducive environment for cell proliferation and differentiation, thereby expediting bone reconstruction.

The technique utilized in clinical practice to accelerate osteogenesis through periosteum construction is commonly referred to as the membrane-induced osteogenesis technique, also known as the Masqueret technique [53]. This method involves the initial insertion of a bone cement spacer into the bone defect during the initial surgical phase, followed by the formation of a bioactive membrane around it [54]. Subsequently, in the second surgical phase, the spacer is extracted and the bone defect site is filled with graft material. The aforementioned method has exhibited considerable promise in addressing critical bone defects by facilitating bone regeneration through the initial development of a natural periosteum [55]. However, it is evident that this approach is not without challenges, including uncontrolled proliferation of the natural periosteum, the complexity of determining the optimal interval between surgical interventions, the adverse effects of multiple surgeries on patients, and an increased vulnerability to infection [56]. As a result, the utilization of commercialized biomimetic periosteum in addressing severe bone defects has been restricted, aiming to eliminate the necessity for repeated surgeries and promote bone regeneration. Nevertheless, the existing biomimetic periosteum predominantly comprises collagen and demonstrates insufficient mechanical characteristics, thereby presenting difficulties in effectively addressing friction at the site of fracture and compressing the soft tissue encompassing the bone defect region. In our study, the PLLA/HA@Irisin nanofibers demonstrated exceptional mechanical characteristics, including elevated tensile strength and flexibility, rendering it an optimal candidate for bone tissue engineering. Additionally, it served as a membrane for cellular adhesion and proliferation, both of which were crucial for mimicking artificial periosteum. Furthermore, the PLLA/HA@Irisin membranes functioned as a barrier, impeding the infiltration of undesired tissues into the bone defect site, thereby mitigating the risk of soft tissue encroachment. The adjustment of the mechanical properties of the membranes to align with those of the adjacent bone tissue serves to augment the integration of the regenerated tissue with the pre-existing bone, thereby ensuring the requisite long-term stability and functionality of the vascularized bone tissue.

## Conclusion

In the present study, we successfully fabricated PLLA/HA@Irisin nanofibrous membranes using the coaxial electrospinning technique, featuring a core-shell structure composed of irisin-loaded nanofibers. The results demonstrated that the release of irisin from the PLLA/HA@Irisin nanofibers enhanced the osteogenic differentiation of BMMSCs. Mechanistically, PLLA/HA@Irisin membranes improved the mitochondrial function of BMMSCs and protected them from oxidative stress via activating SIRT3 and downstream antioxidant enzymes. *In vivo* experiments revealed that *in situ* implantation of PLLA/HA@Irisin was efficacious in promoting the formation of new bone matrix and blood vessels in critical-sized calvarial defects. Overall, our findings demonstrated the PLLA/HA@Irisin membrane represented a novel biomimetic calvarial periosteum for applications in bone tissue engineering.

## Supplementary Material

rbad096_Supplementary_Data

## Data Availability

The data that support the findings of this study are available from the corresponding author upon reasonable request.
