# Supplementary material for: Irisin-loaded electrospun core-shell nanofibers as calvarial periosteum accelerate vascularized bone regeneration by activating the mitochondrial SIRT3 pathway
Source: Regen Biomater. 2023 Oct 31;11:rbad096. doi: 10.1093/rb/rbad096 (PMC10761201; doi:10.1093/rb/rbad096)
Supplement: rbad096_Supplementary_Data [file rbad096_supplementary_data.docx]

**Supplementary Figures**


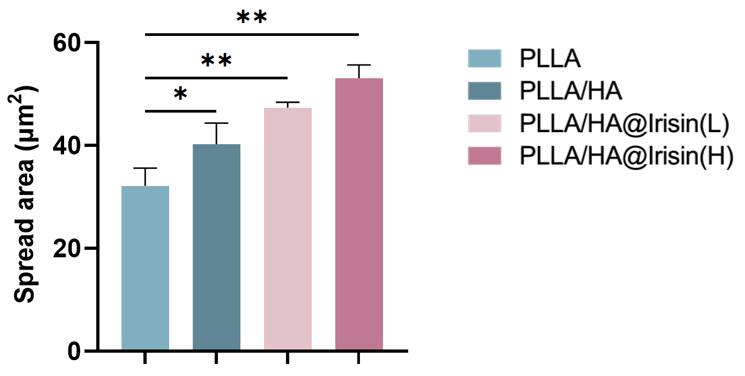


**Supplementary Fig. 1.** The quantification of cell spreading area, n=3. Statistically significant differences were indicated by * *P* < 0.05 or ** *P* < 0.01.

**
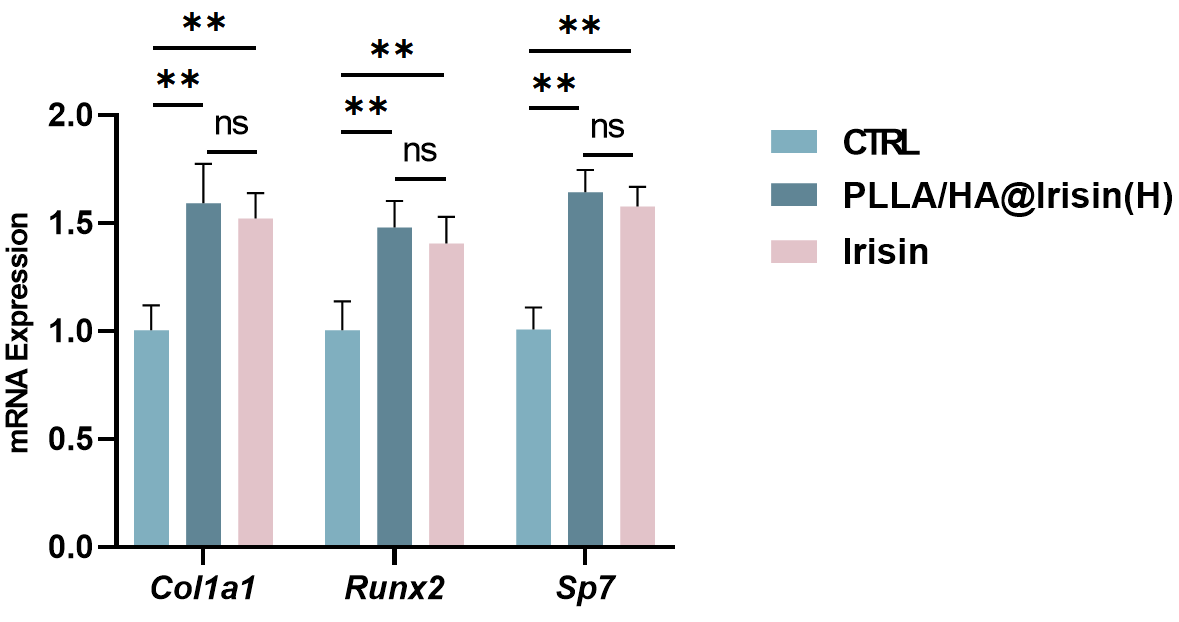
**

**Supplementary Fig. 2.** The gene expression of osteogenic makers, including *Col1a1*, *Sp7*, and *Runx2*, was determined by RT-PCR, n=4. Statistically significant differences were indicated by * *P* < 0.05 or ** *P* < 0.01.


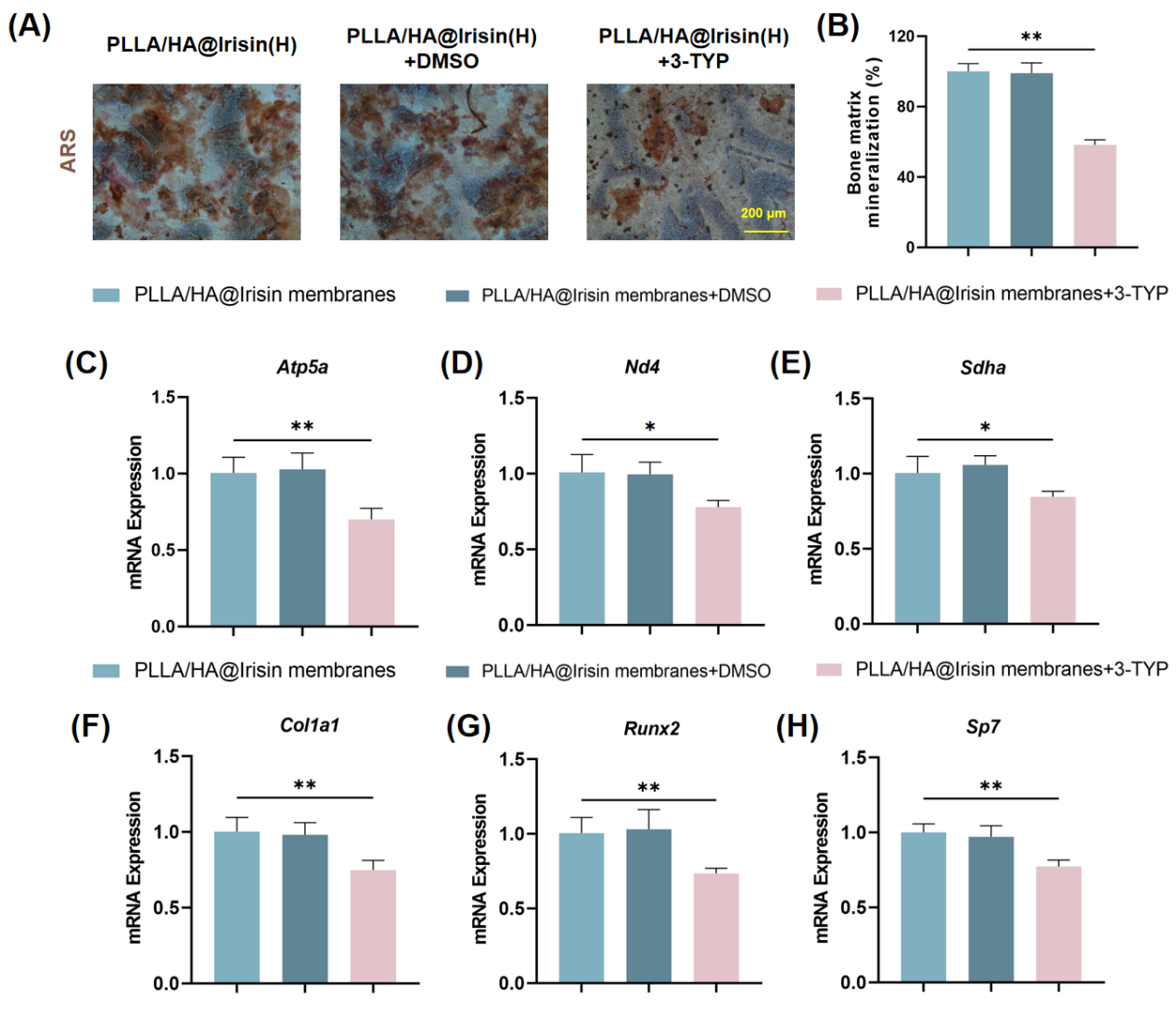


**Supplementary Fig. 3.** The role of PLLA/HA@Irisin membranes in promoting osteogenic differentiation and mitochondrial enhancement was weakened by 3-TYP. (A) Representative images of bone mineral deposition in BMMSCs stained by ARS. Scale bar = 200 μm. (B) Quantification of the stained bone mineral deposition in BMMSCs cultured on different groups of membranes, n=3. (C-E) The gene expression of mitochondrial respiratory chain factors, including *Atp5a*, *Nd4*, and *Sdha*, was determined by quantitative RT-PCR, n=4. (F-H) The gene expression of osteogenic makers, including *Col1a1*, *Sp7*, and *Runx2*, was determined by RT-PCR, n=4. Statistically significant differences were indicated by * *P* < 0.05 or ** *P* < 0.01.
